# The phenotypic variance gradient – a novel concept

**DOI:** 10.1002/ece3.1298

**Published:** 2014-10-20

**Authors:** Cino Pertoldi, Jørgen Bundgaard, Volker Loeschcke, James Stuart Flinton Barker

**Affiliations:** 1 Department 18/Section of Environmental Engineering, Aalborg UniversityAalborg, Denmark; 2 Aalborg ZooAalborg, Denmark; 3 Department of Bioscience, Aarhus UniversityAarhus C, Denmark; 4 School of Environmental and Rural Science, University of New EnglandArmidale, NSW, 2351, Australia

**Keywords:** Canalization, *Drosophila aldrichi*, environmental variability, phenotypic plasticity, Taylor's power law, wing traits

## Abstract

Evolutionary ecologists commonly use reaction norms, which show the range of phenotypes produced by a set of genotypes exposed to different environments, to quantify the degree of phenotypic variance and the magnitude of plasticity of morphometric and life-history traits. Significant differences among the values of the slopes of the reaction norms are interpreted as significant differences in phenotypic plasticity, whereas significant differences among phenotypic variances (variance or coefficient of variation) are interpreted as differences in the degree of developmental instability or canalization. We highlight some potential problems with this approach to quantifying phenotypic variance and suggest a novel and more informative way to plot reaction norms: namely “a plot of log (variance) on the y-axis versus log (mean) on the x-axis, with a reference line added”. This approach gives an immediate impression of how the degree of phenotypic variance varies across an environmental gradient, taking into account the consequences of the scaling effect of the variance with the mean. The evolutionary implications of the variation in the degree of phenotypic variance, which we call a “phenotypic variance gradient”, are discussed together with its potential interactions with variation in the degree of phenotypic plasticity and canalization.

## Introduction

### Reaction norms

Recent studies suggest that phenotypic variability can allow rapid adaptation to new conditions (Queitsch et al. 2002) and may represent a bet-hedging strategy that enhances fitness in fluctuating environments (Acar et al. 2008). Several studies of various traits (see e.g. Auld et al. 2010 and references therein) have attempted to obtain more detailed knowledge of the evolutionary role of phenotypic variance (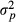) across environments. 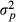 is determined by the interplay of genotypic variance (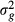), environmental variance (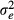), phenotypic plasticity and canalization.

In a sexually reproducing population, the phenotypic variability (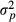) can be given by: 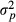 = 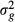 + 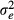 (Falconer and Mackay 1996). The problem with estimating 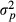 even in a monoclonal strain (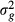 = 0) is that the estimate in general will be strongly affected by 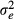 (Pertoldi et al. 2001a,b). If the environmental variance is negligible (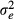 ≈ 0), the phenotypic variance is roughly correlated with the genetic variability (Pertoldi et al. 2003, 2006a,b).

In order to analyze the degree of phenotypic variance and phenotypic plasticity of morphometric and life-history traits, evolutionary ecologists commonly use reaction norms in which the means of traits are plotted against an environmental gradient (DeWitt et al. 1998; Karan et al. 1999). Further, when a set of genotypes is tested over the same gradient, variation among the means at each level of the gradient is a measure of the genetic differences among them.

### Canalization

Canalization is genetic buffering that has evolved under natural selection in order to stabilize the phenotype and decreases its variability (Gibson and Wagner 2000). It can therefore be defined as the ability of systems to withstand genetic or environmental perturbations. In the literature, the terms genetic buffering and canalization are sometimes used as synonyms (see e.g. (Gibson and Wagner 2000; Debat and David 2001; Dworkin 2005). However, these terms are describing different concepts; genetic buffering refers to inherited mechanisms that keep a trait constant and hence decrease the variance about the mean (Dworkin 2005). Whereas canalization is genetic buffering that has evolved under natural selection in order to stabilize the phenotype and decreases its variability (Meiklejohn and Hartl 2002). Canalized traits have an increased capacity to absorb mutational variance. This suggests relatively large genetic variations can be hided in canalized traits with a restricted range of phenotypic variations (Meiklejohn and Hartl 2002).

Wagner et al. (1997) defined two kinds of canalization; environmental and genetic canalization (Dworkin 2005). Environmental canalization is assessed by raising individuals having the same genotype in different environments, whereas genetic canalization is assessed by raising individuals having different genotypes in the same environment (Nijhout and Davidowitz 2003).

Lerner (1954) suggested that multiple heterozygosity in complex multigenic systems provides a mechanism for maintaining plasticity and genetic variability, and for promoting canalization. A low flexibility of developmental pathways may be the outcome of natural selection resulting in high canalization (Gilchrist and Partridge 2001). Canalization can be estimated by measuring 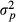 among individuals within an environment (Waddington 1942; Gibson and Wagner 2000). A common way to compare the phenotypic variance of a given trait across different environments is to compare either the 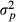 with an *F*-test or to compare the coefficients of variation of the trait in the different environments. Different traits are exposed to different degrees of stabilizing selection and canalization depending on their functional significance. Those traits that are more closely related to fitness are expected to be better buffered against environmental effects (Woods 1999).

### Phenotypic plasticity

A separate term, phenotypic plasticity, has been introduced to describe the effect of different environments on phenotypic expression, which includes changes in behavior, physiology, morphology, growth and life history, and can be expressed either within the lifespan of an individual or across generations (DeWitt et al. 1998; Debat and David 2001; Pigliucci 2005; Kjærsgaard et al. 2007; Krag et al. 2009). Differences among the slopes of reaction norms (the regression of the mean on the environmental gradient) for different traits are interpreted as differences of the degree of phenotypic plasticity (Pigliucci 2005; Krag et al. 2009). Genotype × environment interactions for a trait within populations, which suggest, genetic variation for phenotypic plasticity, will increase the phenotypic variance of the traits, which will depress the response to a selective pressure (Pigliucci 2005; Pertoldi and Bach 2007).

### Taylor's Power law

As mentioned above, 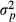 of a trait gives an indication of the degree of canalization. However, there is a biological tendency for 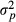 to scale proportionally to the square of the mean (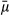):


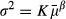
(1)

where *K* is a measure of individual level variability, and *β* is the scaling exponent, which is equal to 2 (see Pertoldi et al. 2007, 2008 for derivation). Consequently, the regression of log *σ*^2^ (dependent) on log 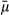 (independent) gives a line with a slope of 2 and this positive relationship between *σ*^2^ and 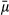 is called Taylor's power law (Taylor 1961; Mutsunori 1995), which means that 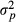 scales proportionally to the square of 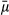, as also pointed out by Mutsunori (1995).

Both log transformation of the variance (Neves et al. 2012), Box–Cox power transformation (Ronnegard and Valdar 2011) and the coefficient of variation (Levy and Siegal 2008) have often been used to compare variation among traits (see Geiler-Samerotte et al. 2013).

However, this approach can result in misleading conclusions, as either can be correctly used only if *β* = 2 (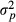 must scale proportionally to the square of 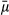) (Taylor and Woiwod 1980). However, 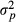 in many morphological traits has been found to scale proportionally to 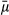 itself, rather than the square of 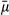 (e.g. Fisher 1937; Yablokov 1974). Another case where 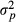 has been shown not to scale proportionally to the square of 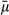 has been discussed by Bader and Hall (1960), who showed that the CV of a composite measure is always less than the weighted average of the CVs of its parts. That is, if trait *X* is composed in reality of two distinct compartments *A* and *B*, that is *X* = *A* + *B*, then the variance of *X* will be:


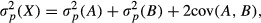
(2)

where 2cov(*A*,*B*) = 2*rσ*_*p*_(*A*)*σ*_*p*_(*B*), and r is the correlation coefficient between trait *A* and trait *B*. Hence, if traits *A* and *B* are negatively correlated, 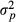
(*X*) will be deflated by this correlation and 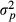 will not scale proportionally to the square of 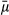 but to an exponent below 2, whereas if r is positive, then 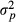 will scale with an exponent higher than 2. In all these cases, however, if 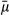 is different at different levels of the environmental gradient, then the CV, the variance and the log of the variance are all inappropriate measures for comparing 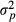 at the different levels of the environmental gradient.

### Aim of the investigation

Our aim was to highlight some of these problems with this approach to quantifying differences in phenotypic variance (i.e. using the CV, or the variance, or the log of the variance when comparing the 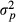 of a trait across an environmental gradient) and to suggest a novel method to plot these reaction norms. This latter approach gives an immediate impression of how the degree of phenotypic variance varies across environments, taking into account the consequences of the scaling effect of 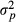 with 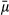 across an environmental gradient. We also introduce the concept of “phenotypic variance gradient” and discuss its evolutionary implications.

## Material and Methods

### Experimental design

We used laboratory-reared progeny (males and females) of wild-caught flies (*Drosophila aldrichi*), originating from five populations in Queensland, Australia, spanning an 800 km transect. The progeny of these five populations was reared at three different temperatures (20°C, 25°C and 30°C) at controlled densities (see Loeschcke et al. 2000 for full details of the methods).

The proximal length of the third longitudinal wing vein (L3p), the distal length of the third longitudinal vein (L3d) and wing width 1 (W1) of 840 flies (420 males and 420 females) were measures with negligible measurement error (see Loeschcke et al. 2000 for all details). The large sample size of wing measured has been necessary in order to reduce the standard error of the variance, which is dependent of the sample size.

### Statistical analysis

An analysis of differences between the slopes of the reaction norms across the temperature gradient was done to assess qualitatively the differences in the degree of phenotypic variance between traits (within population), between sexes (within population) and in the same trait (between populations). This analysis compared the slopes of the reaction norms for the log-transformed values of 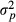 and 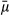 of the traits (between 20°C and 25°C and between 25°C and 30°C) with the “reference line” (slope *β* = 2) (Taylor 1961).

Differences between these slope values are interpreted as differences in the degree of phenotypic variance across the environments. The steeper and more positive the slope, the faster is the degree of phenotypic variance increasing from one environment to another (in our study from one temperature to another, for example from 20°C to 25°C). Where the slope is negative, the degree of phenotypic variance decreases across environments. The extent to which the degree of phenotypic variance changes across environments will be called the “phenotypic variance gradient”, which can be measured as the difference in regression coefficients. Our data set was tested for differences between the slopes measured for a trait in the interval (between 20°C and 25°C and between 25°C and 30°C) by comparing the values of the slopes of the regressions by means of a *Z*-test within a locality (Zar 1999). Furthermore, all the traits' slopes were tested for significant deviations from the expected value of *β* = 2.

In order to get an idea on the effect of the standard error of the variance, we conducted a simulation by generating two groups of values of normally distributed points with the same means and different variance. The normal distributions were simulated with different number of points (2, 3, 4…120) and for every simulation, 100 replications were made. The variances of the simulated distributions were then tested for differences with an *F*-test, and the ratios of the *F*-tests, which result to be significant, were plotted versus the sample size.

All the statistical analyses and graphic plotting were performed using the softwares PAST (Hammer et al. 2001), and the simulations were conducted with the software program StatView SE+Graphics™ (SAS System) (2006).

## Results

**Figure 1:**
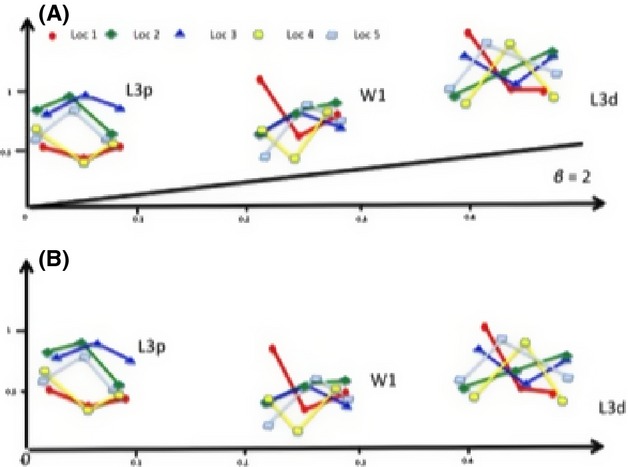
Reaction norms of the log(variance) versus log(mean) for three wing trait measurements: the proximal length of the third longitudinal vein (L3p), the distal length of the third longitudinal vein (L3d) and wing width 1 (W1), in males (Fig. 1A) and females (Fig. 2A) of five populations (LOC 1, LOC 2, LOC 3, LOC 4 and LOC 5) of *D. aldrichi* reared in the laboratory at three different temperatures (30°C, 25°C and 20°C – left to right in figure). All traits were smallest at 30°C and largest at 20°C.

**Figure 2:**
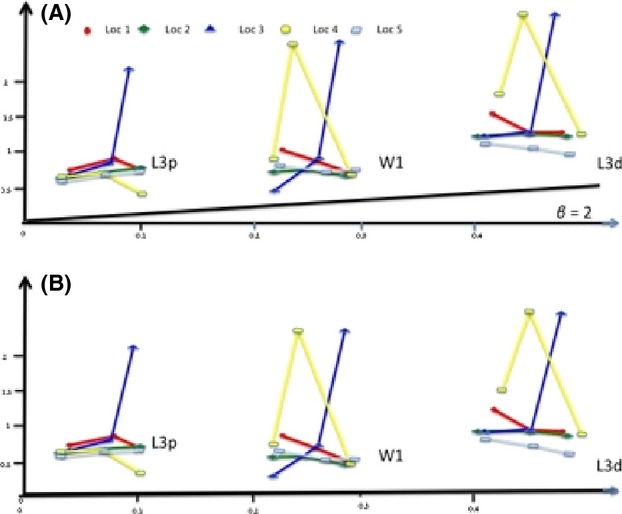
As in Figure 1, but with the slopes of the lines corrected by subtracting the value of 2.

The reaction norms (log variance on log mean – Figs 1A, 2A) give a graphical impression that the degree of phenotypic variance within a population collected in one locality is varying both among traits and at different rearing temperatures. As the 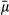 of the traits varied at different rearing temperatures (see Loeschcke et al. 2000), changes of 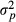 should also vary with a *β* = 2. However, the results in Table 1 show that all the traits (with only four exceptions), deviate significantly (*P* < 0.05) from this expected relationship, with *β* values ranging from −38.2 to 95.75 (26 negative *β* values (*β* < 0) and 34 values of *β* > 0. Table 1 also shows that even within a single locality, *β* values vary significantly (*P* < 0.05) between traits (with only five exceptions), when compared between the two temperature intervals. The *β* values of a given trait also vary between sexes and between populations collected in the five localities (see Table 1). In addition, the fact that all the traits investigated deviated from this expected relationship imply also that the slopes of the regressions shown in (Figs 1A, 2A) need to be corrected by subtracting from all the slopes the values of 2. In Figs 1B, 2B, the corrected slopes have been plotted. After this correction, the slopes which had a slope >2 in Figs 1A, 2A continue to remain positive (slope >0) even if the slope is reduced (corrected slope = uncorrected slope − 2). If the values of the slopes in Figs 1A, 2A are (0< slope <2), the sign of the slope will become negative in (Figs 1B, 2B). Lastly if the slopes in (Figs 1A, 2A) are negative (slope <0), then the slope will become even more negative in (Figs 1B, 2B).

**Table 1:** Values of the slopes of the proximal length of third longitudinal vein (L3p), the distal length of third longitudinal vein (L3d) and wing width 1 (W1) for males and females of five populations of *Drosophila aldrichi* reared at three different temperatures (20°C, 25°C and 30°C)

Locality		1	1	2	2	3	3	4	4	5	5
Temperature comparisons		20–25	25–30	20–25	25–30	20–25	25–30	20–25	25–30	20–25	25–30
Males											
L3p	*β*	**6.1**	−5	−7.9	**7.5**	−7.3	**7.6**	−12.2	−7.2	−7.9	**7.7**
L3d	*β*	**(0)**	−12.3	7.5*	7.5*	**8.3**	−6.4	−11.5	**16.8**	−6.4	**16.8**
W1	*β*	**7.6**	−12.5	**4.9**	**7.7**	−6.1	**7.7**	**16.7**	−7.7	−7.4	**16.5**
Females											
L3p	*β*	−7.6	**8.1**	8.1*	8.1*	**95.7**	**7.7**	−7.6	**6**	6.6*	6.4*
L3d	*β*	**(0)**	−7.7	−6.7	**6.8**	**95.4**	**6.9**	−37.9	**93.1**	−6.8*	−6.9*
W1	*β*	−7.5*	−7.4*	−5.5	**(2.1)**	**95.1**	**16.8**	−38.2	**95.5**	**(2.1)**	−4.9

The values of the slopes >2 are in bold. The traits, which did not show significant differences between the slopes in the two temperatures intervals (20–25°C and 25–30°C) within the same locality, are marked with an asterisk (*); the traits, which did not show significant deviations from the expected value of *β* = 2, are in parentheses.

**Figure 3:**
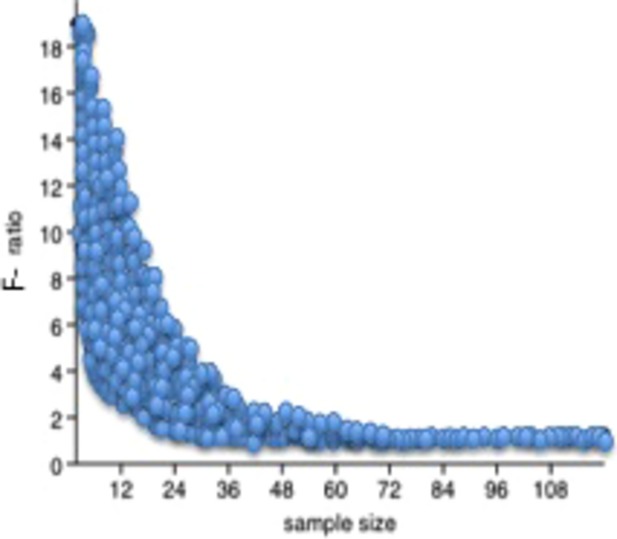
Plot of the significant *F*-ratio values (at the *P* < 0.05 level) versus the sample size (*n*, from 2 to 120) of the simulated normal distributions (100 replications for each sample size).

The results of the simulations conducted in order to quantify the effects of the standard error of the variance shows that a sample size of *n* = 30 allows us to detect a significant differences between two variances when one variance is the half of the other, whereas with *n* = 60, an *F*-ratio of *F* = 1.5 is enough to detect a significant difference at the (*P* < 0.05 level (Fig. 3).

## Discussion

The 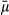 of the traits, which varied among populations and between sexes, was found to be negatively correlated with temperature (Loeschcke et al. 2000), in accordance with numerous previous studies (see Loeschcke et al. 2000 and references therein). The change in size of the traits implies a consequent change in the expected 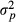 at different temperatures. However, the *β* values were heterogeneous, varying among traits, populations, temperatures and sexes, so that the *β* values deviated from the predicted values (*β* = 2).

The differences among the *β* values when measuring the same trait in the same sex in the different populations can still be used for comparative analysis. The reaction norms presented by Loeschcke et al. (2000) show differences in the degree of phenotypic plasticity, as there are differences in slope between the trait mean values measured at different temperatures. Differences in phenotypic plasticity have evolutionary implications. Selection can in fact only work on differences in phenotypes, and phenotypic plasticity can reduce the correlation between genotype and phenotype. Therefore, phenotypic plasticity can increase the short-term ability to respond to environmental changes, but can also reduce the potential for long-term changes (Sultan 1996; Pertoldi et al. 2005; van Buskirk and Steiner 2009). Fitness traits may be expected to show canalization in a variable environment, showing a stability to environmental perturbations (Liefting et al. 2009).

However, the novel way in which the reaction norms showed in Fig. 2A, B corrected by subtracting the value of two from all the slopes shown in Figs 1A, 2A can give information useful for predicting the changes in the degree of evolutionary potential across an environmental gradient.

We have in fact shown that without the suggested corrections, we are prone to wrongly interpret the changes of the degree of phenotypic variance across environment. We illustrate three examples:

1. When looking for example at the log mean and the log variance of one of the traits which is varying in mean between the two different temperatures (Figs 1A, 2A) and estimating the slope between these two temperatures, if you observe a slope bigger than 2 (slope >2) that you will conclude that the degree of phenotypic variance has been reduced at the temperature in which the trait is largest. This conclusion is not wrong from a qualitative point of view, but it is wrong from the quantitative point of view as the degree to which the phenotypic variance has been reduced is overestimated, and therefore, it has to be corrected by subtracting the value of two from the estimated slope.
2. If the slope of the trait has a value between 0 and 2 (0< slope <2), then we commit both a qualitative and a quantitative error. In fact, will still conclude that the degree of canalization is smaller at the temperature in which the trait is largest compared to the temperature where the trait is smaller. However, this is not true because after a correction of the slope by subtracting the value of 2, the slope will become negative, which means that the degree of phenotypic variance is in reality higher at the temperature where the trait is largest compared to the temperature where the trait is smaller. And the correct value of the negative slope can be estimated again by subtracting the value of 2.
3. If the slope of the trait is negative, then the error will not be qualitative but quantitative as the interpretation that the degree of phenotypic variance for the trait is higher at the temperature in which the trait is largest compared to the degree of canalization at the temperature in which the trait is smaller, is not wrong. However, the degree at which phenotypic variance has increased when moving from one temperature to the other has been underestimated as the slope has to be corrected with a subtraction of the value of 2, making the slope even more negative.

The degree of phenotypic variance of a trait is often represented in a reaction norm with a vertical bar (symmetric around the 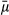 of the trait which can represent the standard deviation, the standard error or the confidence interval). However, from these graphical representations, it is not possible to get an accurate quantification of the degree of phenotypic variance and/or an accurate estimation of the degree of increase or decrease of the phenotypic variance between the environments, as the estimates of phenotypic variance are not corrected for the scaling effect. Hence, we emphasize the specific way in which the reaction norms have been presented here, and how it makes possible estimation of a “phenotypic variance gradient”. The “phenotypic variance gradient” concept can be utilized to predict whether the evolutionary potential will increase (slope >2) or decrease (slope <2) when moving, for example, from one temperature to another or across any kind of environmental gradient. The degree of phenotypic variance of a trait can in fact affect its capacity to evolve. This is due to the fact that the capacity of a trait to respond to selection is reduced by any process that reduces the level of expressed variation of the trait.

The heterogeneity of the slope values observed for the different traits in Figs 1A and 2A indicates that these traits will be exposed to different selection regimes when environmental conditions change. Also, the differences observed for the slopes when measured for the same trait between the two sexes, indicates that the selective regimes also will vary between sexes when environmental conditions change. This novel way to plot and correct the phenotypic variance of a trait is opening new perspective in the field of quantitative genetics and in the associated studies. Several techniques: proteomic tools, techniques for studying the function of noncoding small RNAs, next-generation sequencing have been applied with the attempt to discover the molecular and cellular mechanisms of phenotypic plasticity and canalization (Cote et al. 2007; Aubin-Horth and Renn 2009). These studies will clearly benefit if an unbiased estimate of the phenotypic variance can be obtained. The results of the simulations presented in this study show that a large sample size is necessary for small differences in variances to be statistically significant
